# Supplementary material for: A deep learning approach to private data sharing of medical images using conditional generative adversarial networks (GANs)
Source: PLoS One. 2023 Jul 6;18(7):e0280316. doi: 10.1371/journal.pone.0280316 (PMC10325103; doi:10.1371/journal.pone.0280316)
Supplement: S1 Fig — Column A illustrates an example of a pairwise attack of a synthetic dataset. The green digit from candidates is closely matching the green digit from the synthetic dataset meaning this candidate is likely in the trainset and privacy is not preserved. Column B illustrates an example of a distribution attack on a synthetic dataset. While not being an exact match, the green digit is very similar to the digits from synthetic. This indicates that the green digit is likely present in the training set. (PDF) [file pone.0280316.s001.pdf]

S1 Fig. Candidate pair definition

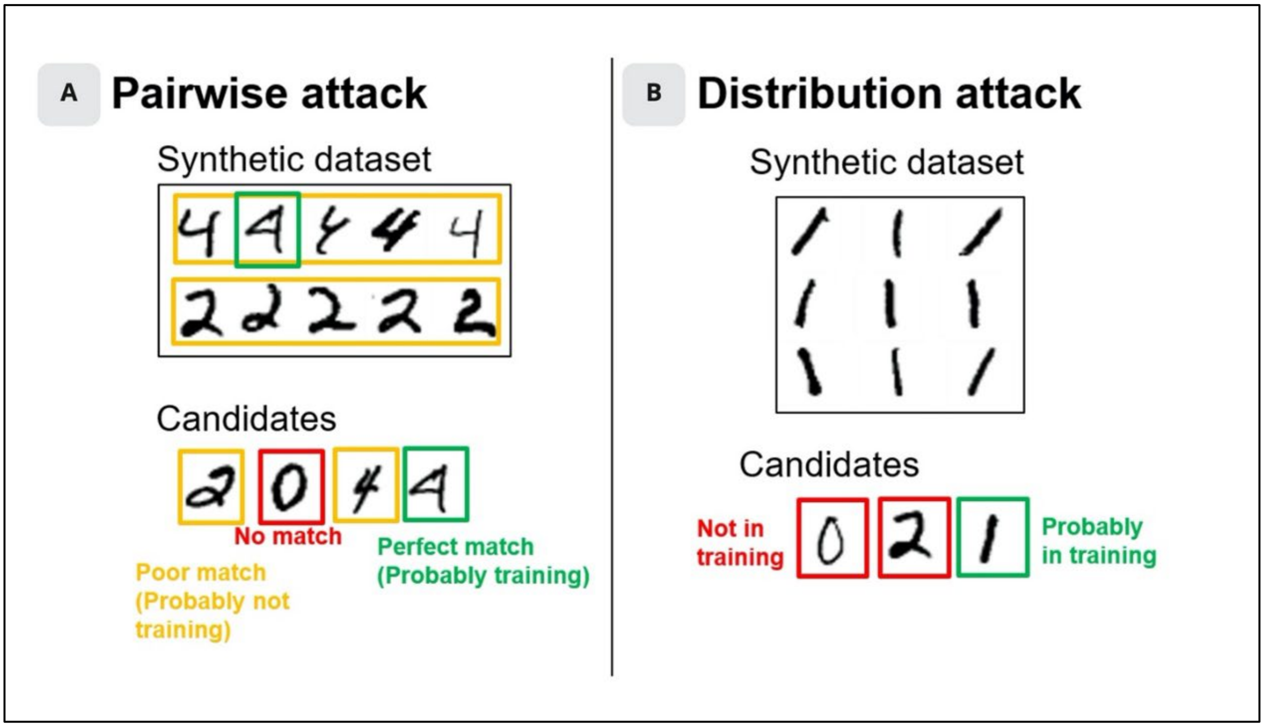

Column A illustrates an example of a pairwise attack of a synthetic dataset. The green digit from candidates is closely matching the green digit from the synthetic dataset meaning this candidate is likely in the trainset and privacy is not preserved. Column B illustrates an example of a distribution attack on a synthetic dataset. While not being an exact match, the green digit is very similar to the digits from synthetic. This indicates that the green digit is likely present in the training set.
