# Supplementary material for: A deep learning approach to private data sharing of medical images using conditional generative adversarial networks (GANs)
Source: PLoS One. 2023 Jul 6;18(7):e0280316. doi: 10.1371/journal.pone.0280316 (PMC10325103; doi:10.1371/journal.pone.0280316)
Supplement: S2 Fig — In this experiment, the abnormally low distances between candidates and synthetic images can be identified by anomaly detection. An abnormal sample is any sample that diverges largely from the distance test-synthetic (green distribution). For the synthetic dataset, validation samples are also classified as outliers, privacy was preserved. (PDF) [file pone.0280316.s002.pdf]

S2 Fig. Privacy attack scenarios

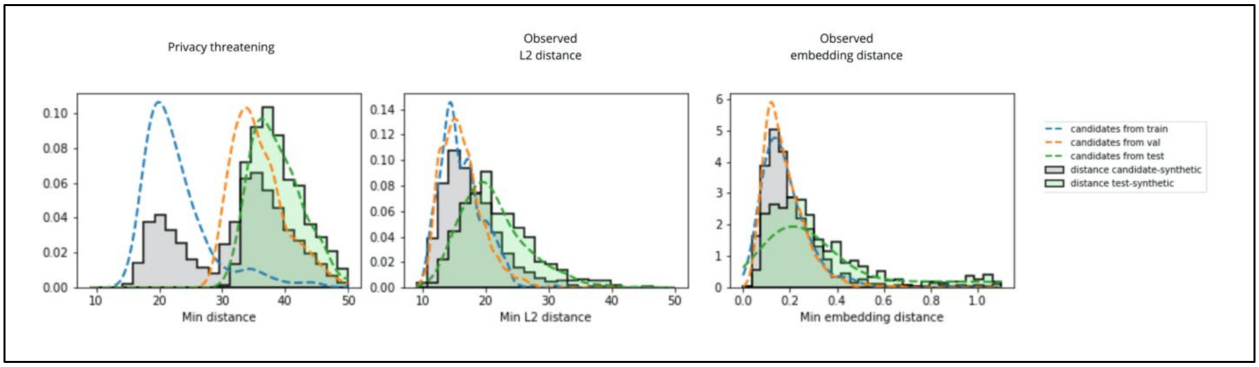

In this experiment, the abnormally low distances between candidates and synthetic images can be identified by anomaly detection. An abnormal sample is any sample that diverges largely from the distance test-synthetic (green distribution). For the synthetic dataset, validation samples are also classified as outliers, privacy was preserved.
