# Supplementary material for: A deep learning approach to private data sharing of medical images using conditional generative adversarial networks (GANs)
Source: PLoS One. 2023 Jul 6;18(7):e0280316. doi: 10.1371/journal.pone.0280316 (PMC10325103; doi:10.1371/journal.pone.0280316)
Supplement: S3 Fig — ROC for classification of candidate samples from train vs validation (orange) and train vs test (green) are presented. A) Pairwise attacks. The classification is under the assumption candidates with the lowest distances are likely from training. B) Distribution attacks. The classification is done by associating large clusters to training samples. A high AUC means it is easy to classify training from other samples. (PDF) [file pone.0280316.s003.pdf]

## S3 Fig. ROC curves

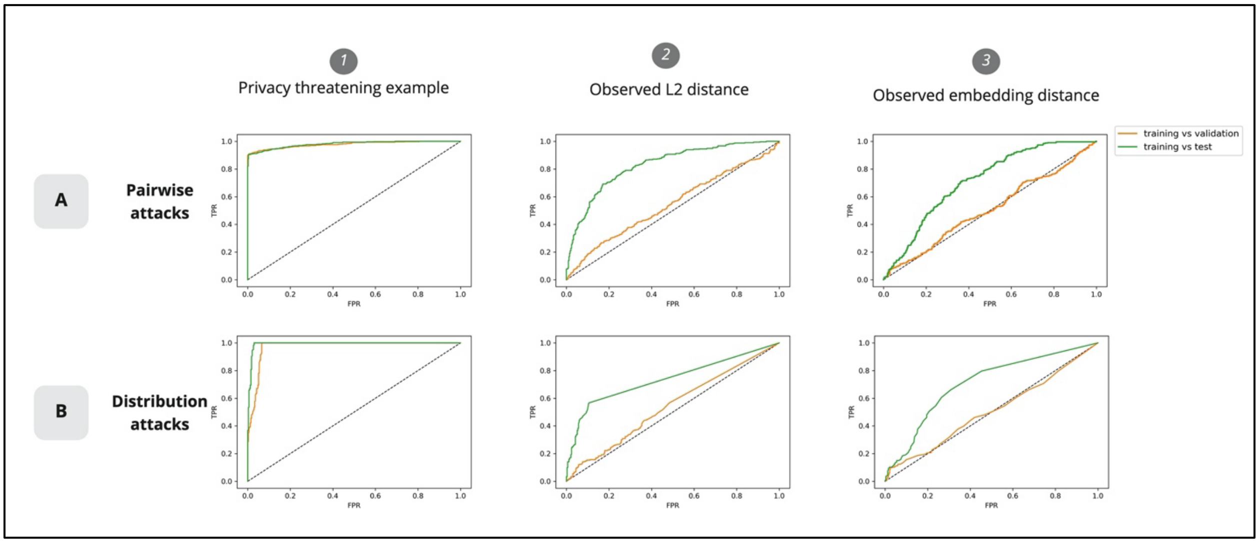


ROC for classification of candidate samples from train vs validation (orange) and train vs test (green) are presented. A) Pairwise attacks. The classification is under the assumption candidates with the lowest distances are likely from training. B) Distribution attacks. The classification is done by associating large clusters to training samples. A high AUC means it is easy to classify training from other samples.
