# Supplementary material for: A deep learning approach to private data sharing of medical images using conditional generative adversarial networks (GANs)
Source: PLoS One. 2023 Jul 6;18(7):e0280316. doi: 10.1371/journal.pone.0280316 (PMC10325103; doi:10.1371/journal.pone.0280316)
Supplement: S4 Fig — The cut-off is defined as the threshold for a point to be considered an outlier. The orange curve shows the proportion of outliers from training vs outliers from validation. The green curve shows the proportion of outliers from training vs outliers from test. A privacy threatening case corresponds to almost all the outliers coming from the training set. (PDF) [file pone.0280316.s004.pdf]

S4 Fig. Cut-off curves

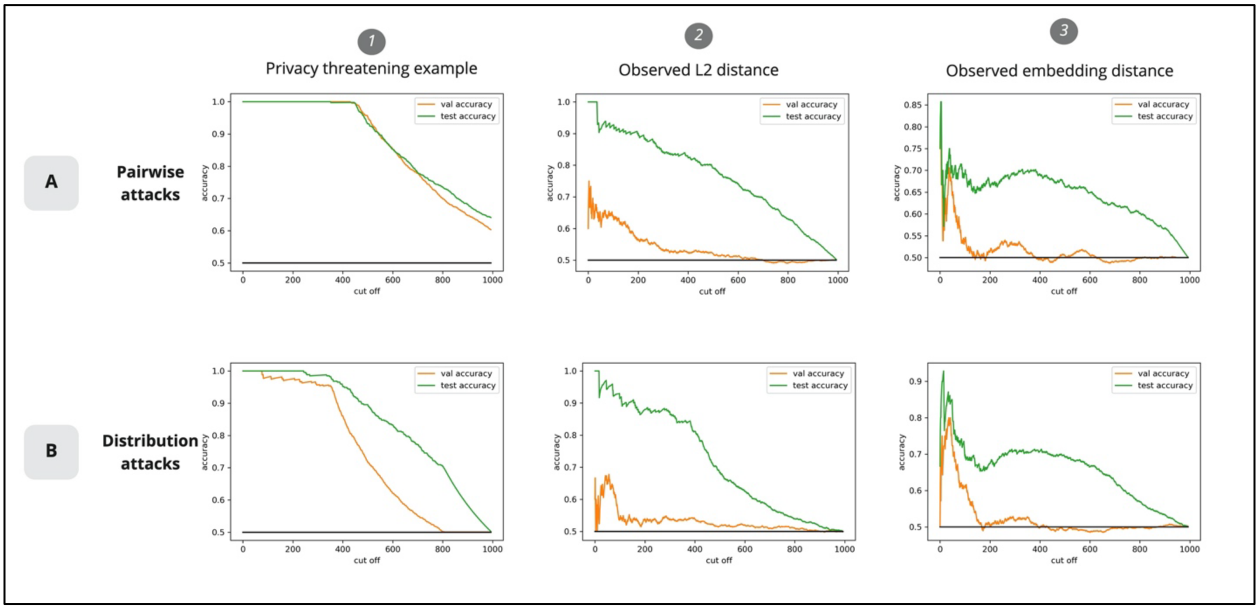

The cut-off is defined as the threshold for a point to be considered an outlier. The orange curve shows the proportion of outliers from training vs outliers from validation. The green curve shows the proportion of outliers from training vs outliers from test. A privacy threatening case corresponds to almost all the outliers coming from the training set.
