# Supplementary material for: A deep learning approach to private data sharing of medical images using conditional generative adversarial networks (GANs)
Source: PLoS One. 2023 Jul 6;18(7):e0280316. doi: 10.1371/journal.pone.0280316 (PMC10325103; doi:10.1371/journal.pone.0280316)
Supplement: S1 Table — Proportion of origin for different candidates for a fixed cut-off. Cut-off 50 means the top 50 largest clusters or smallest distances are considered outliers (50 is top 5% of the 1000 candidate images). In extreme privacy threatening scenarios, it was expected that all training sample can be identified as outliers, meaning they would represent 100% of the top 333 candidates. (PDF) [file pone.0280316.s005.pdf]

S1 Table. Classification of candidate origin

	Pairwise attacks					
	Toy example		L2 distance		Embedding distance	
**Cut-off**	**50**	**333**	**50**	**333**	**50**	**333**
Train proportion	1.00	0.89	0.58	0.48	0.51	0.43
Val proportion	0.00	0.04	0.36	0.43	0.27	0.38
test proportion	0.00	0.07	0.05	0.09	0.22	0.19

	Distribution attacks					
	Toy example		L2 clusters		Embedding clusters	
**Cut-off**	**50**	**333**	**50**	**333**	**50**	**333**
Train proportion	0.93	0.91	0.64	0.49	0.62	0.43
Val proportion	0.05	0.07	0.33	0.42	0.24	0.39
Test proportion	0.02	0.02	0.04	0.09	0.15	0.18

Proportion of origin for different candidates for a fixed cut-off. Cut-off 50 means the top 50 largest clusters or smallest distances are considered outliers (50 is top 5% of the 1000 candidate images). In extreme privacy threatening scenarios, it was expected that all training sample can be identified as outliers, meaning they would represent 100% of the top 333 candidates.
