# Supplementary material for: A deep learning approach to private data sharing of medical images using conditional generative adversarial networks (GANs)
Source: PLoS One. 2023 Jul 6;18(7):e0280316. doi: 10.1371/journal.pone.0280316 (PMC10325103; doi:10.1371/journal.pone.0280316)
Supplement: Attachment — Submitted filename: Response to reviewers.docx [file pone.0280316.s006.docx]

**Responses to editorial comments:**

When submitting your revision, we need you to address these additional requirements.

**We thank the editor for the feedback. We have edited the manuscript accordingly.**

1. Please ensure that your manuscript meets PLOS ONE's style requirements, including those for file naming. The PLOS ONE style templates can be found at

<https://journals.plos.org/plosone/s/file?id=wjVg/PLOSOne_formatting_sample_main_body.pdf> and [https://journals.plos.org/plosone/s/file?id=ba62/PLOSOne_formatting_sample_title_authors_affiliations.pdf](https://urldefense.com/v3/__https:/journals.plos.org/plosone/s/file?id=ba62*PLOSOne_formatting_sample_title_authors_affiliations.pdf__;Lw!!N3hqHg43uw!oZb65GXEPOQ1DBAeTQNgMsI4S00jvTG5ACJXQw1UWitv7bcwLFMq9GLDn6LWtFMmhWeUpcj6pHvXtf3olPiY28c$)

**We have made the necessary formatting changes as per the template provided.**

2. Please provide in your manuscript text information on the location of the A2209 dataset, either as a literature reference or as a URL link to the location of the dataset.

**We have added reference in the manuscript line 122** - Baeten *et al.* [Anti-interleukin-17A monoclonal antibody secukinumab in treatment of ankylosing spondylitis: a randomised, double-blind, placebo-controlled trial](https://www.sciencedirect.com/science/article/pii/S0140673613611344?via%3Dihub). Lancet 2013

3. We note that the grant information you provided in the ‘Funding Information’ and ‘Financial Disclosure’ sections do not match.

When you resubmit, please ensure that you provide the correct grant numbers for the awards you received for your study in the ‘Funding Information’ section.

**We have added “Novartis Pharma AG” as the funder in the appropriate section in the submission site**

4. Thank you for stating the following in the Competing Interests:

“I have read the journal's policy and HS, AJ, TK declared no competing interests.

I have read the journal's policy and TC, MB, JP, SS, GL, AR, DO are employees of
Novartis.”

We note that one or more of the authors have an affiliation to the commercial funders of this research study : Novartis

a. Please provide an amended Funding Statement declaring this commercial affiliation, as well as a statement regarding the Role of Funders in your study. If the funding organization did not play a role in the study design, data collection and analysis, decision to publish, or preparation of the manuscript and only provided financial support in the form of authors' salaries and/or research materials, please review your statements relating to the author contributions, and ensure you have specifically and accurately indicated the role(s) that these authors had in your study. You can update author roles in the Author Contributions section of the online submission form.

Please also include the following statement within your amended Funding Statement.

“The funder provided support in the form of salaries for authors [insert relevant initials], but did not have any additional role in the study design, data collection and analysis, decision to publish, or preparation of the manuscript. The specific roles of these authors are articulated in the ‘author contributions’ section.”

If your commercial affiliation did play a role in your study, please state and explain this role within your updated Funding Statement.

b. Please also provide an updated Competing Interests Statement declaring this commercial affiliation along with any other relevant declarations relating to employment, consultancy, patents, products in development, or marketed products, etc.

Within your Competing Interests Statement, please confirm that this commercial affiliation does not alter your adherence to all PLOS ONE policies on sharing data and materials by including the following statement: "This does not alter our adherence to  PLOS ONE policies on sharing data and materials.” (as detailed online in our guide for authors <http://journals.plos.org/plosone/s/competing-interests>). If this adherence statement is not accurate and  there are restrictions on sharing of data and/or materials, please state these. Please note that we cannot proceed with consideration of your article until this information has been declared.

Please include both an updated Funding Statement and Competing Interests Statement in your cover letter. We will change the online submission form on your behalf.

**We have included the updated funding statement and competing interest statement in the revised cover letter**

5. PLOS requires an ORCID iD for the corresponding author in Editorial Manager on papers submitted after December 6th, 2016. Please ensure that you have an ORCID iD and that it is validated in Editorial Manager. To do this, go to ‘Update my Information’ (in the upper left-hand corner of the main menu), and click on the Fetch/Validate link next to the ORCID field. This will take you to the ORCID site and allow you to create a new iD or authenticate a pre-existing iD in Editorial Manager. Please see the following video for instructions on linking an ORCID iD to your Editorial Manager account: <https://www.youtube.com/watch?v=_xcclfuvtxQ>

**We have linked the corresponding author’s ORCID ID with the manuscript**

6. Thank you for stating the following in the Acknowledgments Section of your manuscript:

“The authors thank the study investigators for their contributions. The studies were funded by Novartis Pharma AG, Basel, Switzerland, in accordance with Good Publication Practice (GPP3) guidelines (<http://www.ismpp.org/gpp3>). “

We note that you have provided additional information within the Acknowledgements Section that is not currently declared in your Funding Statement. Please note that funding information should not appear in the Acknowledgments section or other areas of your manuscript. We will only publish funding information present in the Funding Statement section of the online submission form.

Please remove any funding-related text from the manuscript and let us know how you would like to update your Funding Statement. Currently, your Funding Statement reads as follows:

“The study was sponsored by Novartis Pharma AG. Novartis personnel and academic advisors from Oxford Big Data Institute (BDI) designed the project.”

Please include your amended statements within your cover letter; we will change the online submission form on your behalf.

**We have removed the funding statement from Acknowledgement and included the funding statement and competing interest statement in the revised cover letter**

7. Please amend your manuscript to include your abstract after the title page.

**Abstract has been included in the manuscript**

8. Please include captions for your Supporting Information files at the end of your manuscript, and update any in-text citations to match accordingly. Please see our Supporting Information guidelines for more information: <http://journals.plos.org/plosone/s/supporting-information>.

**Captions for Supporting Information has been updated and added at the end of the manuscript.**

**Reviewer's Responses to Questions**

We thank the reviewer for the constructive feedbacks. We edited the manuscript accordingly to them.

1. Lack of large enough dataset preventing investigation of full spine images instead of vertebral units.

We agree with the reviewer. The lack of data for full synthetic MRIs was the main reason why we focused on vertebras units (VUs) instead of whole images. By using VUs, we increased our effective sample size by pooling all time steps and VU locations together. We acknowledge this in the Limitations (line 421). Following-up on the comment, we added further comments on the issue (line 422).

We acknowledged the data limitation to generate 21 vertebras units (VUs) at once (i.e., a whole spine) at line 447 in the Discussion.

2. Difficulty to use the pGAN on clinical metrics which is clinically important.

We added further detail on these results. To follow good science practices, we shared negative results from this experiment as well as successful one (i.e., VU location). We also added our interpretation of why the clinical conditioning failed as opposed to the location. We edited the manuscript accordingly in the Limitation (line 428)

3. It is also important to note that despite several tested scenarios the presented study does not provide any mathematical guarantees for the privacy of the synthetic data, and there are likely cases in which privacy would be breached in practice.

We tested the most common scenario under clear condition. The manuscript does not make a claim for a mathematically guaranteed privacy model, but rather demonstrates how the model was tested under real scenarios (pairwise and distribution attacks).

4. Assessment of fidelity is not presented in a reader study, just by one figure.

We agree with the reviewer. The fidelity was indeed assessed by generating synthetic images. We generated many images and presented it internally. While this approach is by no means meant to quantify their fidelity, it gave us a high confidence on their visual quality. We then exhaustively tested our images to train a model from scratch (diversity) to ensure that they are representative and informative.

5. Why aren't the training and validation datasets from F2305 study and test dataset from A2209 study divided by 23 VUs of each patient? How were the numbers of VUs generated?

We thank the reviewer for this question. The number of samples is counted as VUs and not as patient because VUs were treated as independent of their spinal location to increase our effective sample size. We edited the manuscript accordingly in the methods and discussion.

6. How many patients were used for the test datasets?

The independent test set (A2209) included 27 patients. We edited the manuscript line 122 to reflect this information. We added the reference to the manuscript as well: Baeten *et al.* [Anti-interleukin-17A monoclonal antibody secukinumab in treatment of ankylosing spondylitis: a randomised, double-blind, placebo-controlled trial](https://www.sciencedirect.com/science/article/pii/S0140673613611344?via%3Dihub). Lancet 2013.

7. guess Fig 2 in the text is fig 5 in the actual images, Fig 3/4/5 in the text are Fig 2/3/4? – please revise.

We thank the reviewer for this comment and apologize with the mistake done during submission time. We changed the figure numbers and edited the text accordingly.

8. guess that cases with spinal hardware have been excluded?

During the collection of the MRI scans, patients with pacemakers, aneurysm clips, artificial heart valves, ear implants, metal fragments or foreign objects in the eyes, skin or body that rendered the patient unable to undergo MRI were excluded from submitting MRI scans, therefore patient scans utilized in this analysis are not likely to include spinal hardware.
